# Activation of Coagulation and Fibrinolysis in Acute Respiratory Distress Syndrome: A Prospective Pilot Study

**DOI:** 10.3389/fmed.2016.00064

**Published:** 2016-11-28

**Authors:** Agnese Ozolina, Marina Sarkele, Olegs Sabelnikovs, Andrejs Skesters, Inta Jaunalksne, Jelena Serova, Talis Ievins, Lars J. Bjertnaes, Indulis Vanags

**Affiliations:** ^1^Department of Cardiac Surgery, Pauls Stradins Clinical University Hospital, Riga, Latvia; ^2^Riga Stradins University, Riga, Latvia; ^3^Department of Anesthesiology and Intensive Care Unit, Pauls Stradins Clinical University Hospital, Riga, Latvia; ^4^Laboratory of Biochemistry, Riga Stradins University, Riga, Latvia; ^5^Clinical Immunology Centre, Pauls Stradins Clinical University Hospital, Riga, Latvia; ^6^Anesthesia and Critical Care Research Group, Department of Clinical Medicine, Faculty of Health Sciences, University of Tromsø, Tromsø, Norway

**Keywords:** acute respiratory distress syndrome, lung injury, plasminogen activator inhibitor-1, tissue factor, tissue plasminogen activator, ventilator-associated lung injury

## Abstract

**Introduction:**

Coagulation and fibrinolysis remain sparsely addressed with regards to acute respiratory distress syndrome (ARDS). We hypothesized that ARDS development might be associated with changes in plasma coagulation and fibrinolysis. Our aim was to investigate the relationships between ARDS diagnosis and plasma concentrations of tissue factor (TF), tissue plasminogen activator (t-PA), and plasminogen activator inhibitor-1 (PAI-1) in mechanically ventilated patients at increased risk of developing ARDS.

**Materials and methods:**

We performed an ethically approved prospective observational pilot study. Inclusion criteria were patients with PaO_2_/FiO_2_ < 300 mmHg admitted to the intensive care unit (ICU) for mechanical ventilation for 24 h, or more, because of one or more disease conditions associated with increased risk of developing ARDS. Exclusion criteria were age below 18 years; cardiac disease. We sampled plasma prospectively and compared patients who developed ARDS with those who did not using descriptive statistics and chi-square analysis of baseline demographical and clinical data. We also analyzed plasma concentrations of TF, t-PA, and PAI-1 at inclusion (*T*_0_) and on third (*T*_3_) and seventh day (*T*_7_) of the ICU stay with non-parametric statistics inclusive their sensitivity and specificity associated with the development of ARDS using receiver operating characteristic curve analysis. Statistical significance: *p* < 0.05.

**Results:**

Of 24 patients at risk, 6 developed mild ARDS and 4 of each moderate or severe ARDS, respectively, 3 ± 2 (mean ± SD) days after inclusion. Median plasma concentrations of TF and PAI-1 were significantly higher at *T*_7_ in patients with ARDS, as compared to non-ARDS. Simultaneously, we found moderate correlations between plasma concentrations of TF and PAI-1, TF and PaO_2_/FiO_2_, and positive end-expiratory pressure and TF. TF plasma concentration was associated with ARDS with 71% sensitivity and 100% specificity, a cut off level of 145 pg/ml and AUC 0.78, *p* = 0.02. PAI-1 displayed 64% sensitivity and 100% specificity with a cut off concentration of 117.5 pg/ml and AUC 0.77, *p* = 0.02. t-PA did not change significantly during the observation time.

**Conclusion:**

This pilot study showed that increased plasma concentrations of TF and PAI-1 might support ARDS diagnoses in mechanically ventilated patients after 7 days in ICU.

## Introduction

Acute respiratory distress syndrome (ARDS) represents a severe respiratory failure characterized by hypoxia and non-hydrostatic pulmonary edema with bilateral opacities on frontal chest radiographs. Patients with ARDS require treatment in the intensive care unit (ICU) for respiratory support with continuous positive airway pressure (CPAP) or positive end-expiratory pressure (PEEP) of 5 cmH_2_O or higher in combination with mechanical ventilation (1–3). Usually, ARDS occurs within 1 week of a triggering event (4, 5) and might be the result of a direct lung injury, as pneumonia, or an indirect lung injury, like septic shock (6–8).

Incidence of ARDS displays large geographical differences (9, 10). A recent worldwide multicenter study showed that 10.4% of those admitted to participating ICUs and 23.4% of those requiring mechanical ventilation exhibited ARDS criteria. Hospital mortality varied among 34.9, 40.3, and 46.1% for patients with, respectively, mild, moderate, and severe ARDS (11). This is consistent with mortality rates published by previous investigators (10, 12). Thus, despite a varying incidence, overall mortality from ARDS has not changed substantially during the last decade (13, 14). Consequently, identification of patients at risk should be prioritized with the aim to diagnose ARDS earlier, as recently proposed (15).

Several studies have focused on injury to the lung parenchyma, which then causes deteriorated gas exchange in ARDS (16–18). In contrast, changes in homeostasis of coagulation and fibrinolysis have been sparsely investigated despite that their role in the evolution of this disease is beyond doubt. Investigators recently described a role in the interaction between lung inflammation and the coagulation and the fibrinolytic systems for the commencement of ARDS (19, 20).

Inflammation modulates blood coagulation by activating C-reactive protein, which stimulates monocytes and alveolar macrophages to generate tissue factor (TF) (5, 21), and endothelial cells to produce plasminogen activator inhibitor-1 (PAI-1) (22). The combined effects can lead to disseminated intravascular coagulation (DIC) including formation of intravascular micro-thrombi and intra-alveolar fibrin deposits (5, 22–24), subsequently increasing both dead-space ventilation and intrapulmonary shunting, both characteristic features of ARDS (23). Consequently, we speculated that the circulatory level of TF, and the balance between inhibitors and activators of fibrinolysis, could be of help for the diagnosing of ARDS (21).

In mechanically ventilated patients, whom we considered to be at increased risk of developing ARDS, we aimed to perform a pilot study to explore whether associations exist between diagnosis of ARDS and the plasma concentration of TF as the primary endpoint, and ARDS and plasma levels of PAI-1 and tissue plasminogen activator (t-PA), respectively, as secondary endpoints.

## Materials and Methods

### Patients Eligible for the Study

Between November 2014 and February 2015, we transferred 33 patients to the ICU of Pauls Stradins Clinical University Hospital (PSCU), Riga, Latvia, for mechanical ventilation because we considered them to be at increased risk of developing ARDS. After the Medical Ethics Committee of PSCU had approved the study protocol and the informed consent form (No. 18204-5 L), we screened the patients for suitability to participate in a prospective observational pilot study of the influence of plasma coagulation and fibrinolysis on the emergence of ARDS. We obtained informed consent from each one’s legal guardian prior to inclusion for those who were eligible for participation in the study.

According to their disease conditions, the patients were treated either with non-invasive pressure-controlled mechanical ventilation (NIV), or endotracheal intubation and mechanical ventilation with CPAP ≥5 cmH_2_O, or PEEP ≥5 cmH_2_O over 24 h or more using Servo-I ventilators (Maquet Getinge, Group, Rastatt, Germany). The patients received antibiotics, as required, based on the results of microbiological cultures and antibiotic resistance testing.

### Inclusion Criteria

We included patients with PaO_2_/FiO_2_ < 300 mmHg and with an increased risk of developing ARDS (23) because of one or more ARDS predisposing conditions causing either direct or indirect lung injury. The former included pneumonia or aspiration of gastric contents into the airways; the latter comprised of sepsis, acute pancreatitis, DIC, burns, drug overdose or trauma with circulatory shock, and massive blood transfusions, as outlined by previous investigators (23). The patients did not fulfill all the required ARDS criteria at the time of inclusion (2). Those who fulfilled ARDS criteria within 1 week of the stay in ICU were randomized to an ARDS group, the others to a non-ARDS group.

We divided those who fulfilled the clinical, radiological, and cardiologic criteria of ARDS into mild, moderate, or severe ARDS with the characteristics listed below. Mild ARDS: 200 mmHg < PaO_2_/FiO_2_ ≤ 300 mmHg, CPAP or PEEP ≥5 cmH_2_O; moderate ARDS: 100 mmHg < PaO_2_/FiO_2_ ≤ 200 mmHg, PEEP ≥5 cmH_2_O; and severe ARDS: PaO_2_/FiO_2_ ≤ 100 mmHg, PEEP ≥5 cmH_2_O, according to the definition (2).

### Exclusion Criteria

We excluded patients below 18 years of age, patients receiving mechanical ventilation with no ARDS predisposing condition, patients not fulfilling the study protocol, and patients from whom we did not get consent for participation. We also excluded patients after transthoracic echocardiography (ECHO) displaying ejection fraction (EF) <50% and right ventricular systolic pressure >35–40 mmHg, and with a pulmonary artery occlusion pressure >18 mmHg, as measured if a Swan Ganz catheter was in place.

### Study Protocol

We determined plasma concentrations of TF, t-PA, and PAI-1 at enrollment (*T*_0_) and on the third day (*T*_3_) and the seventh day (*T*_7_) of the ICU stay. Moreover, we noticed ventilator settings including FiO_2_, tidal volume, airway plateau pressure, and PEEP-levels that were titrated by means of the transpulmonary pressure–volume curve displayed on the Servo-I ventilator, and the resulting readings of PaO_2_/FiO_2_ and lung mechanical parameters from every subject at the time of collection of plasma samples. We also recorded number of days on mechanical ventilation, ventilator-free days at day 30 of the stay in ICU, and the 30-day mortality rate.

We observed the patients in ICU for 1 week and compared plasma concentrations of coagulation and fibrinolysis biomarkers between those who developed ARDS and those who did not develop the disease (non-ARDS), as based on recent criteria (2). Demographic and clinical data and direct and indirect ARDS risk factors were retrieved from the medical records of the patients. The severity of disease was scored at inclusion using the sequential organ failure assessment (SOFA) and the acute physiology and chronic health evaluation (APACHE II) scores. In addition, we calculated lung injury prediction score (LIPS) for every patient at the day of inclusion (*T*_0_).

### Determination of Biomarkers of Coagulation and Fibrinolysis

We collected blood from a peripheral vein into heparin tubes that we cool centrifuged (ELMI CM-6MT^®^, USA) for 5 min at 3,000 × *g*. The plasma supernatant was removed from the spun samples and frozen at −70°C until the time of analysis. Human TF (CD264) with a normal range of from 12.5 to 400 pg/ml was quantitatively assessed by means of an enzyme-linked immunosorbent assay (ELISA) test (Abcam^®^, UK). Fibrinolysis markers, t-PA (normal range 15.6–1,000 pg/ml), and PAI-1 (normal range 78–5,000 pg/ml) were analyzed with the same method using the platinum ELISA kit (eBioscience^®^, USA).

In parallel, we analyzed standard coagulation tests (SCT) such as prothrombin index (PI), international normalized ratio (INR), and fibrinogen plasma concentration, as well as platelet count (PLT), which was analyzed by means of a Beckman Coulter LH 750 Hematology Analyzer (Beckman Coulter International SA, Switzerland). Coulter LH 750 uses impedance technology to measure PLT count (normal range 150–450 × 10^9^/l). Fibrinogen plasma concentration was analyzed in citrated plasma (Multifibren U reagent, Siemens Healthcare Diagnostics, USA). The reference value is 1.8–3.6 g/l. PI was analyzed using a prothrombin complex assay (Lyophilized Dade^®^ and Innovin^®^ reagent, Siemens Healthcare Diagnostics, USA). PI normal range is 70–120%. APTT was analyzed in citrated human plasma (Pathrombin*SL reagent, Siemens Healthcare Diagnostics, USA). APTT normal range is 26–36 s.

### Statistical Analysis and Sample Size Calculations

Statistical analyses were performed using the SPSS Statistics (Chicago, IL, USA, Version 20). We compared ARDS patients and non-ARDS patients and survivors and non-survivors of the disease. Data distribution was assessed using Shapiro–Wilk test or Kolmogorov–Smirnov test, as appropriate. We used descriptive statistics for analysis of baseline demographics and clinical data, and Chi-square or Mann–Whitney rank sum test to compare the difference between groups. If the *F* value was greater than critical, repeated measures ANOVA was followed by Holm–Sidak’s *post hoc* test for pairwise multiple comparisons to check for intragroup differences. For non-normally distributed data, the results were presented as boxes with median and interquartile range (IQR; 25th–75th percentile), including vertical error bars for the 10 and 90% lowest and highest values, respectively. Correlations were presented as Spearman’s *r*_s_ (25). We performed receiver operating characteristic (ROC) curve analysis of coagulation and fibrinolytic biomarkers including calculations of area under the curve (AUC) for TF and PAI-1 to demonstrate their efficiency in supporting ARDS diagnosis. Spearman’s correlation coefficient also was calculated to analyze the relationships between coagulation/fibrinolysis markers and ventilation parameters. Statistical significance was defined as *p* < 0.05. When required, we calculated sample sizes needed to reach significant differences (*p* < 0.05) in plasma concentrations of TF, PAI-1, and t-PA at a power of 80% between the ARDS group and the non-ARDS group.

## Results

### Clinical Course

**Figure 1:**
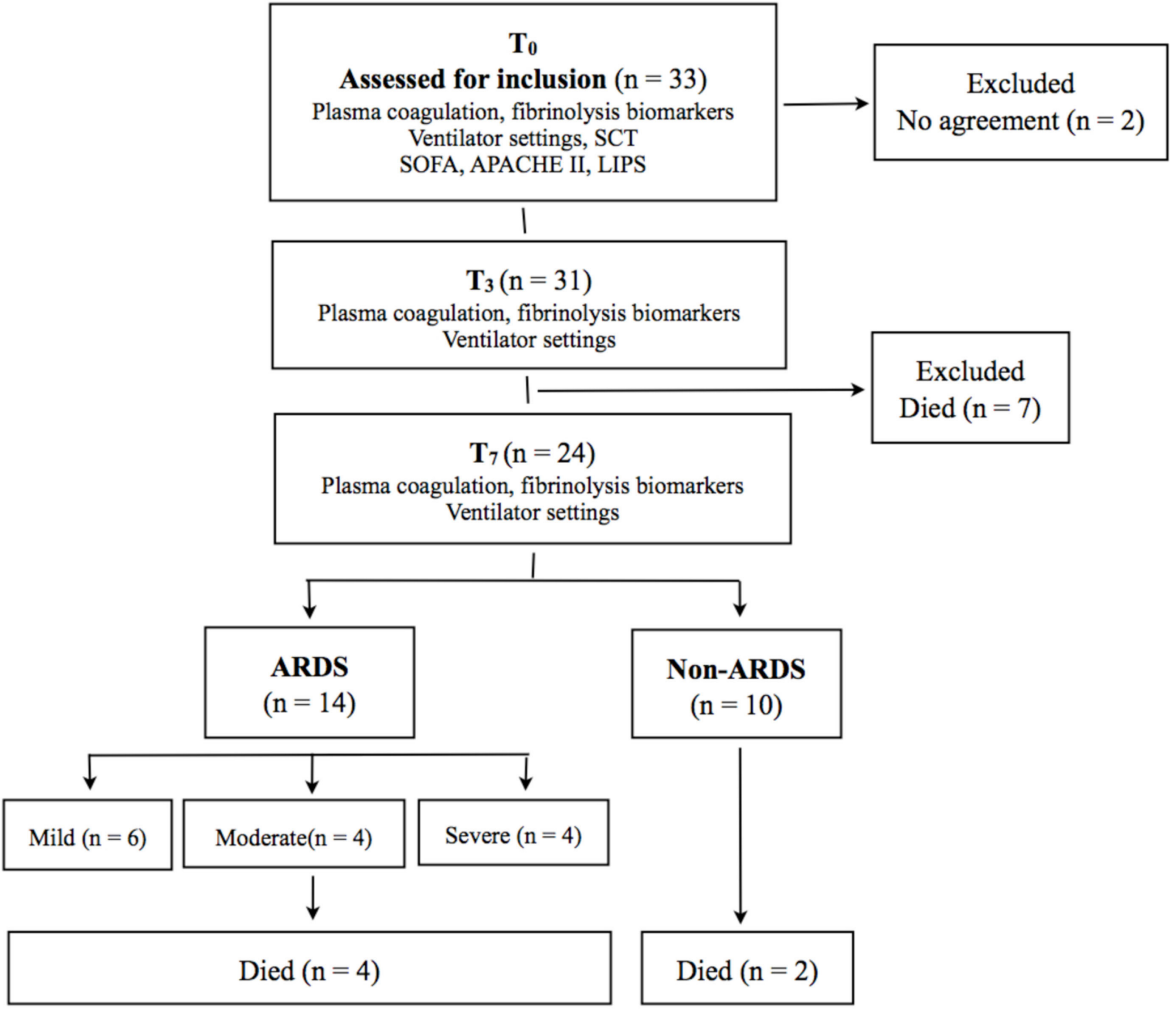
**Mechanically ventilated patients considered for eligibility to a prospective pilot study**. Twenty-four patients fulfilled the study protocol. SCT, standard coagulation tests; APACHE II, acute physiology and chronic health evaluation II; SOFA, sequential organ failure assessment; LIPS, lung injury prediction score; ARDS, acute respiratory distress syndrome.

As depicted in Figure 1, we assessed 33 mechanically ventilated patients, considered to be at risk of developing ARDS, for suitability to the study. We excluded seven patients who died before they fulfilled all the requirements of the study protocol, and two patients from whom we did not get consent for participation.

Table 1 displays demographic characteristics and clinical variables of 24 patients (21 men and 3 women) fulfilling inclusion criteria and all requirements of the protocol encompassing comorbidities, conditions predisposing for ARDS, severity scores, and standard coagulation variables. Pneumonia (46%) was the most common underlying disease. However, neither ARDS nor non-ARDS patients displayed significant intergroup differences with regard to demographic data, comorbidities, or predisposing conditions. Patients diagnosed with ARDS had significantly higher baseline values of SOFA, APACHE II, and LIPS scores. Moreover, the ARDS group showed significant differences in systemic coagulation (elevated INR, lower PLT, and fibrinogen plasma levels), but no clinical signs of DIC. The mean time for developing ARDS was 3 ± 2 days after inclusion. Six patients developed mild ARDS, whereas moderate or severe ARDS occurred within 1 week of the inclusion in four patients each, thus constituting in all 58% of the population at risk. Days in ICU and ventilator-free days at day 30 displayed no intergroup differences. Total hospital mortality and mortality at day 30, reached 25%, but displayed no significant differences between the groups.

**Table 1:** **Baseline demographic characteristics, ARDS predisposing factors, and mortality**.

Demographic data	Totally, *n* = 24	ARDS, *n* = 14	Non-ARDS, *n* = 10	*p*-Value
Age, years	54 ± 17	55 ± 15	53 ± 19	0.7
Women, *n* (%)	3 (12)	2 (14)	1 (10)	0.8
BMI	26.4 ± 4.6	24.7 ± 2.8	27.2 ± 3.2	0.4
**Comorbidities**				
Hypertension, *n* (%)	7 (29)	4 (29)	3 (30)	0.1
Diabetes mellitus, *n* (%)	4 (17)	2 (14)	2 (20)	0.6
COPD, *n* (%)	3 (12)	1 (7)	2 (20)	0.4
**Conditions predisposing for ARDS**				
Pneumonia, *n* (%)	11 (46)	6 (42)	5 (50)	0.7
Sepsis, *n* (%)	7 (29)	4 (29)	3 (30)	0.9
Pancreatitis, *n* (%)	5 (21)	4 (29)	1 (10)	0.3
Massive transfusions, *n* (%)	1 (4)	0	1 (10)	0.2
**Severity scores**				
SOFA, median (range)	4 (3.4–6.1)	5 (4.6–7.2)	3 (2.1– 4.9)	0.04
APACHE II, median (range)	23 (19–28)	25 (22–28)	21 (19–24)	0.03
LIPS median (range)	5.8 (0.3–13)	8.1 (3–13)	4.9 (0.3–9)	0.01
**Coagulation status**				
INR	1.18 ± 0.4	1.2 ± 0.6	1.12 ± 0.15	0.03
PLT (10^9^/l)	172 ± 56	136 ± 47	213 ± 31	0.003
Fibrinogen (mg/dl)	283 ± 71	261 ± 96	312 ± 63	0.02
**ICU stay and mechanical ventilation**				
ICU days, *n*	12 ± 5	14 ± 7	11 ± 4	0.7
Ventilator-free days, *n*	5 ± 3	4 ± 2	7 ± 4	0.4
**Mortality**				
Hospital, *n* (%)	6 (25)	4 (29)	2 (20)	0.1
30-day, *n* (%)	6 (25)	4 (29)	2 (20)	0.1

*Data are presented as mean ± SD, number (*n*), or median (range)*.

*APACHE II, chronic health evaluation score; ARDS, acute respiratory distress syndrome; BMI, body mass index; ICU, intensive care unit; INR, international normalized ratio; LIPS, lung injury prediction score; n, number of patients; PLT, platelets; %, percent; range, lowest and highest value; SOFA, sequential organ failure assessment score*.

Table 2 displays ventilation parameters and PaO_2_/FiO_2_ at *T*_0_, *T*_3_, and *T*_7_. Tidal volumes were significantly smaller at *T*_0_ and *T*_7_ in ARDS patients, as compared to the non-ARDS group. Despite that plateau pressures were significantly higher in the ARDS group at *T*_3_ and *T*_7_, and PEEP also was significantly raised at the latter time point, these ventilator settings did not prevent a significant fall in PaO_2_/FiO_2_ in comparison with the non-ARDS group.

**Table 2:** **Ventilation variables at inclusion (*T*_0_) and at the third (*T*_3_) and seventh (*T*_7_) day of ICU stay in ARDS and non-ARDS patients**.

Ventilation settings	ARDS, *n* = 14		Non-ARDS, *n* = 10		*p*-Value
	Median	IQR	Median	IQR	
**Tidal volume, l/min**					
*T*_0_	8.6	7.1–9.2	10.1	8.8–11.3	0.01
*T*_3_	7.8	6.7–8.9	9.6	8.9–10.1	NS
*T*_7_	7.1	6.6–7.7	8.7	8.3–9.0	0.002
**Plateau pressure, cmH_2_O**					
*T*_0_	29	22–35	24	21–27	NS
*T*_3_	30	23–36	26	22–30	0.002
*T*_7_	27	21–34	25	20–31	0.012
**PaO_2_/FiO_2_**					
*T*_0_	158.5	42–282	183.2	74–348	NS
*T*_3_	193.5	94–362	234	58–358	NS
*T*_7_	179.5	57–292	284	80–356	0.03
**PEEP, cmH_2_O**					
*T*_0_	7	5–11	5	3–7	NS
*T*_3_	7	5–13	5	4–7	NS
*T*_7_	7	5–13	5	2–6	0.006

*Data are presented as median and interquartile range (IQR)*.

*ARDS, acute respiratory distress syndrome; cmH_2_O, centimeter of water; FiO_2_, fraction of inspired oxygen; kg, kilograms; ml, milliliters; n, number of patients; PaO_2_, partial pressure of oxygen in blood; PEEP, positive end-expiratory pressure; NS, not significant*.

### Comparison of Coagulation and Fibrinolysis in ARDS vs. Non-ARDS Patients

**Figure 2:**
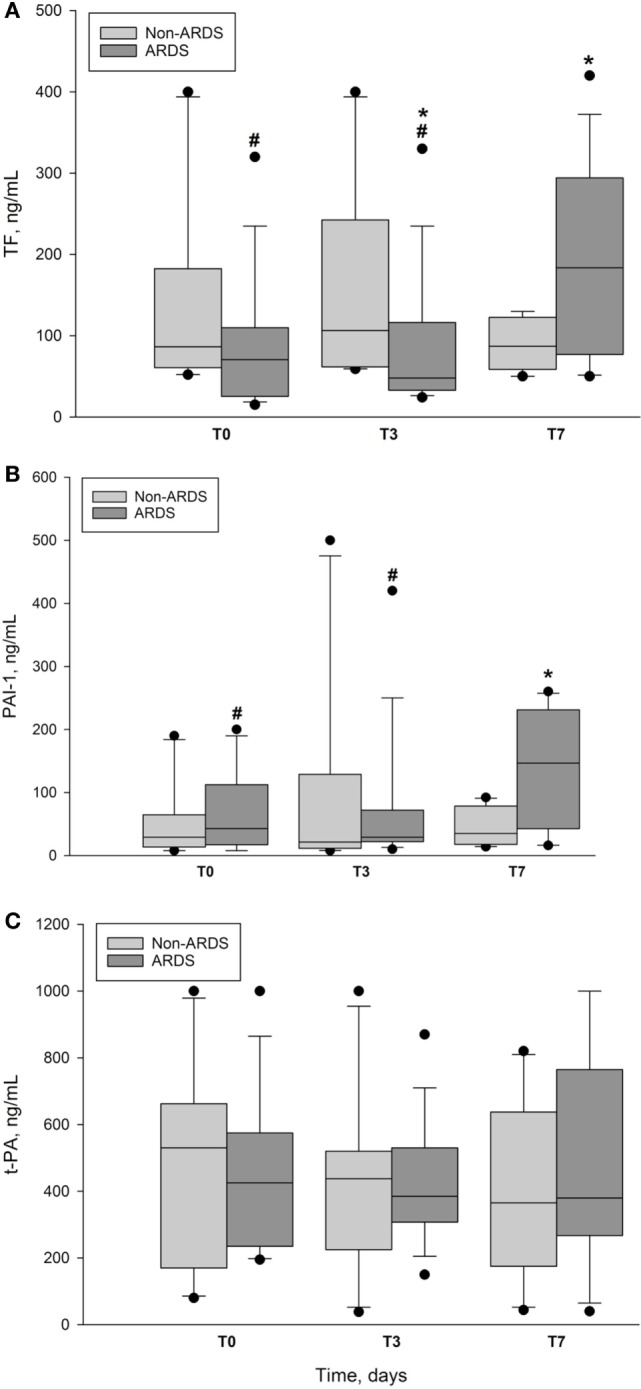
**Plasma concentrations of markers of coagulation and fibrinolysis in ARDS and non-ARDS patients**. **(A)** Plasma concentration of tissue factor (TF) vs. time in ARDS group and non-ARDS group. Data presented as median and interquartile range with 10th and 90th percentiles error bars. *T*_0_ is day of enrollment, *T*_3_ and *T*_7_, the third day and the seventh day of ICU stay. *denotes intergroup *p* = 0.043 at *T*_3_ and *p* = 0.022 at *T*_7_. ^#^denotes ARDS intragroup *p* ≤ 0.001 between *T*_7_ and *T*_0_ and *T*_3_, respectively. pg/ml, picogram per milliliter; closed circles represent outliers. **(B)** Plasma concentrations of plasminogen activator inhibitor-1 (PAI-1) vs. time in ARDS group and non-ARDS group. Data presented as median and interquartile range with 10th and 90th percentiles error bars. *T*_0_ is day of enrollment, *T*_3_ and *T*_7_, the third day and the seventh day of ICU stay. *denotes intergroup *p* = 0.026 at *T*_7_. ^#^denotes ARDS intragroup *p* = 0.016 between *T*_7_ and *T*_0_ and *p* = 0.017 between *T*_7_ and *T*_3_. pg/ml, picogram per milliliter; closed circles represent outliers. **(C)** Plasma concentration of tissue plasminogen activator (t-PA) vs. time in ARDS group and non-ARDS group. Data presented as median and interquartile range with 10th and 90th percentiles error bars. *T*_0_ is day of enrollment, *T*_3_ and *T*_7_, the third day and the seventh day of ICU stay. No significant intergroup or intragroup differences; closed circles represent outliers.

Plasma concentrations of TF (Figure 2A) remained within normal range with no significant difference between ARDS and non-ARDS group at *T*_0_ (*p* = 0.229), but displayed marked intergroup differences at *T*_3_ (*p* = 0.043) and *T*_7_ (*p* = 0.022). The ARDS group also showed intragroup differences in TF plasma concentrations between the highest value at *T*_7_ in comparison with those at *T*_0_ and *T*_3_, respectively (*p* < 0.001). In contrast, we found no significant intragroup differences in the plasma levels of TF in the non-ARDS group.

Plasma levels of PAI-1 (Figure 2B) showed no intergroup differences at *T*_0_ and *T*_3_, but increased at *T*_7_ in the ARDS group, as compared with non-ARDS group (*p* = 0.026). Plasma concentration of PAI-1 at *T*_7_ in the ARDS group was also increased in comparison with intragroup values at *T*_0_ (*p* = 0.016) and *T*_3_ (*p* = 0.017), but not no significant difference was found between the latter concentrations. Plasma levels of t-PA fell at *T*_3_ (Figure 2C), but we noticed no significant differences within or between the groups. At *T*_3_, plasma concentration of t-PA was 295 ± 85 pg/ml (mean ± SD) in ARDS survivors vs. 516 ± 160 pg/ml in ARDS non-survivors, but the difference did not reach significance due to low sample size.

**Figure 3:**
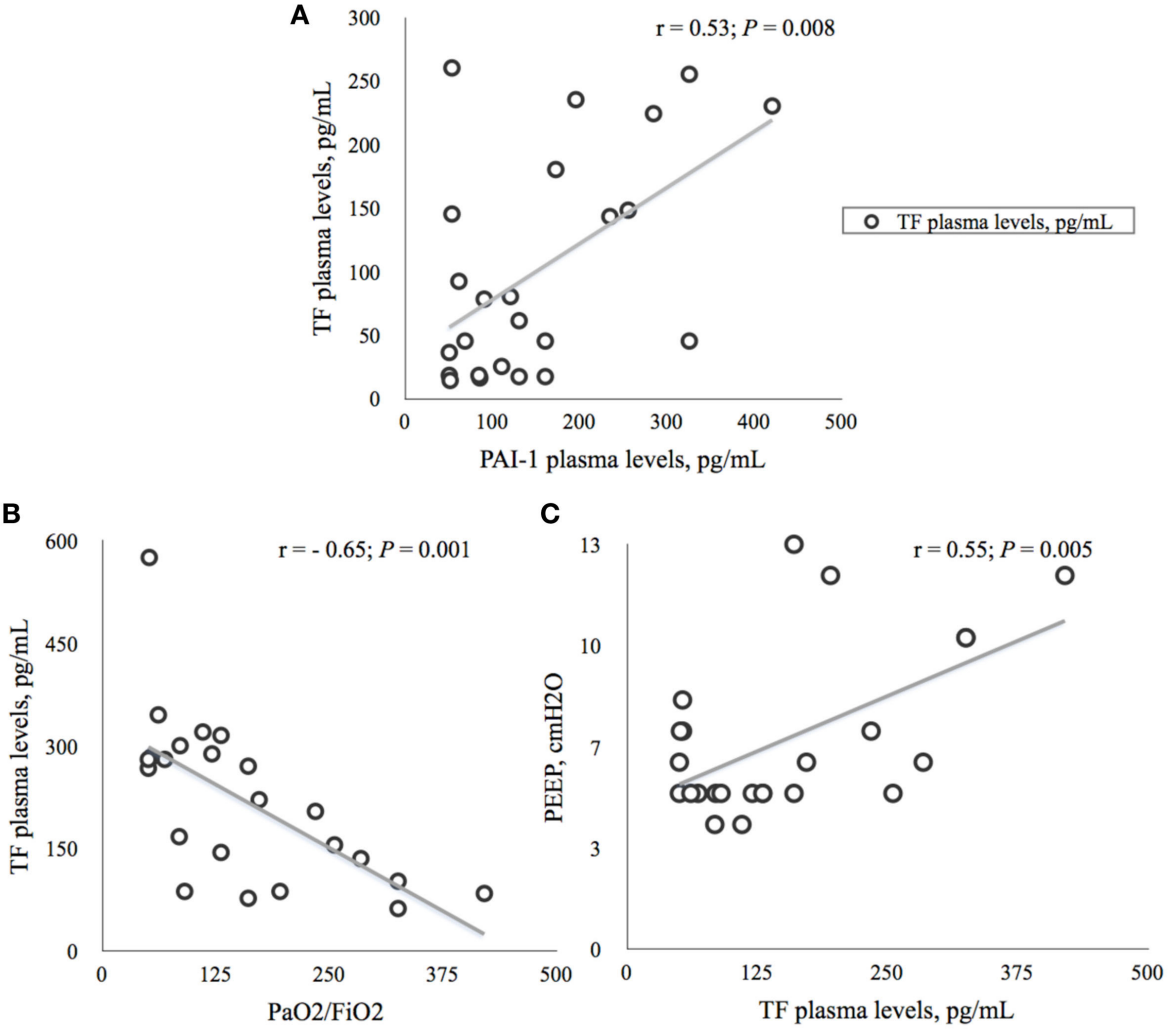
**Correlations between tissue factor (TF) and plasminogen activator inhibitor-1 (AI-1) and ventilation parameters on the seventh day (*T*_7_) of ICU stay**. **(A)** Relationship between plasma concentrations of TF and PAI-1 in 24 ICU patients. Data presented as Spearman’s correlation coefficient (*r*) with *p*-value. TF, tissue factor; PAI-1, plasminogen activator inhibitor; pg/ml, picogram per milliliter. **(B)**. Relationship between plasma concentrations of TF and PaO_2_/FiO_2_ in 24 ICU patients. Data presented as Spearman’s correlation coefficient (*r*) with *p*-value. PaO_2_, arterial oxygen partial pressure; FiO_2_, fraction of inspired oxygen; TF, tissue factor; pg/ml, picogram per milliliter. **(C)** Relationship between plasma concentrations of TF and level of PEEP in 24 ICU patients. Data presented as Spearman’s correlation coefficient with *p*-value. PEEP, positive end-expiratory pressure; cmH_2_O, centimeter of water; TF, tissue factor; pg/ml, picogram per milliliter.

We found significant correlations between plasma concentrations of TF and PAI-1 (*r* = 0.53; *p* = 0.008), TF and PaO_2_/FiO_2_ (*r* = −0.65; *p* = 0.001), and PEEP and TF (*r* = 0.55; *p* = 0.005) at time *T*_7_, as depicted in Figures 3A–C.

**Figure 4:**
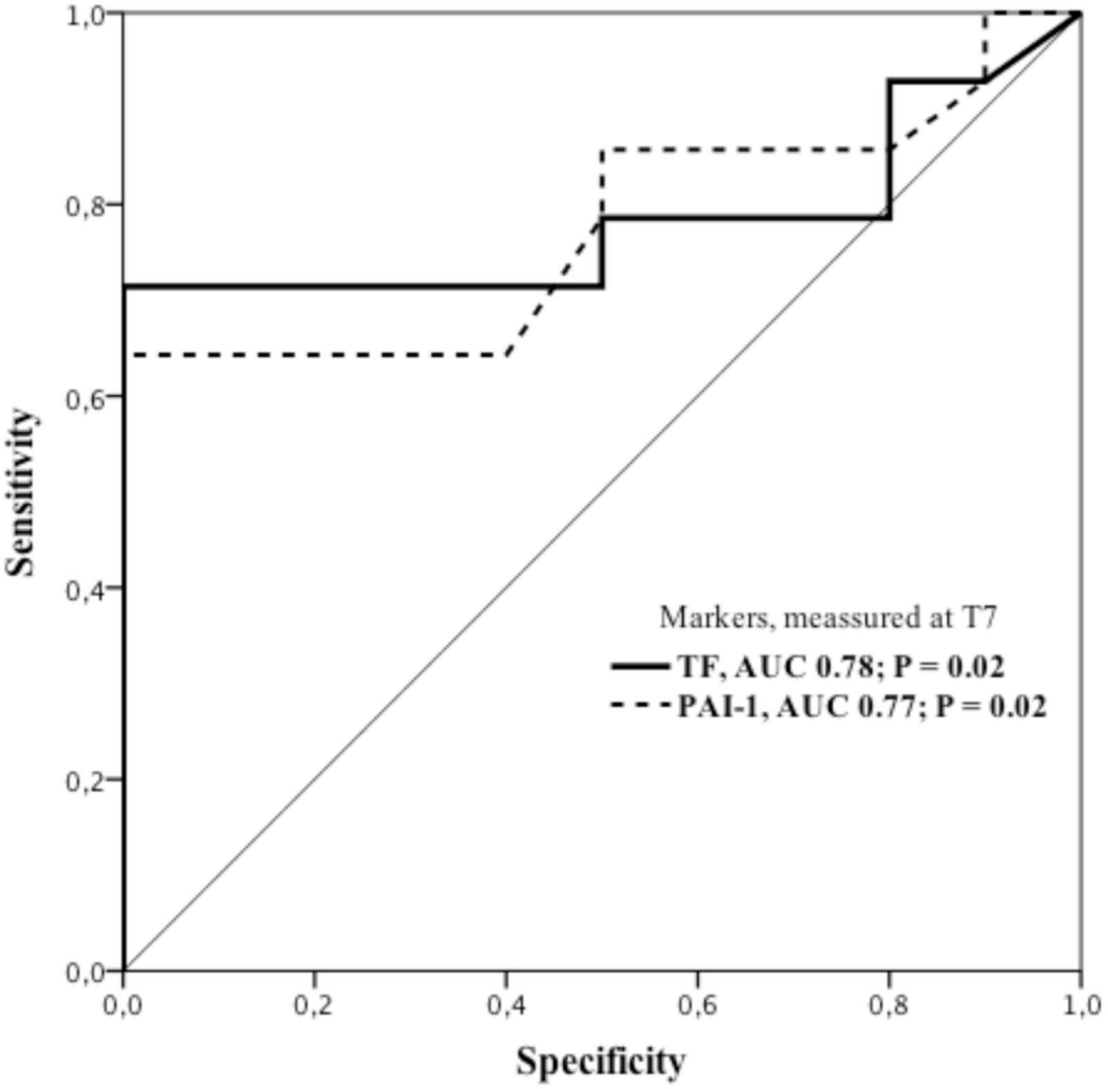
**ROC curves showing sensitivity and specificity of tissue factor and plasminogen activator inhibitor-1 for ARDS diagnoses on the seventh day (*T*_7_) of ICU stay**. ARDS group displaying 71% sensitivity and 100% specificity for association with plasma concentration of TF. Cut off value: 145 pg/ml; AUC 0.782; *p* = 0.021; 95%CI: 0.586–0.979. Correspondingly, plasma concentration of PAI-1 showed sensitivity and specificity of 64 and 100%, respectively, and a cut off value of 117.5 pg/ml; AUC 0.775; *p* = 0.024; 95%CI: 0.584–0.966. AUC, area under the curve; 95%CI, 95% confidence interval; PAI-1, plasminogen activator inhibitor-1; ROC, receiver operating characteristics; TF, tissue factor.

In ARDS patients, a ROC analysis (Figure 4) showed 71% sensitivity and 100% specificity for an association with TF at *T*_7_ with a cut off value of 145 pg/ml [AUC 0.782; *p* = 0.021; 95% confidence interval (95%CI): 0.586–0.979]. We also found an association with PAI-1 determined at the same time point with a sensitivity and specificity of 64 and 100%, respectively, with a cut off value of 117.5 pg/ml (AUC 0.775; *p* = 0.024, 95%CI: 0.584–0.966).

## Discussion

In this prospective pilot study, 14 out of total 24 patients considered to be at increased risk of ARDS, developed the disease within 7 days of commencement of mechanical ventilation. Patients diagnosed with ARDS had significantly higher baseline severity scores in comparison with the non-ARDS group, which could indicate a worse outcome. However, mortality did not differ significantly between groups (Table 1). In the ARDS group, PaO_2_/FiO_2_ reached nadir on the seventh day of the ICU stay, in parallel with significant changes in variables of coagulation and fibrinolysis. In patients diagnosed with ARDS, plasma concentrations of TF and PAI-1 increased significantly at *T*_7_, as compared with the non-ARDS group. The observation of increased TF is consistent with recent findings in patients with sepsis-induced ARDS (5). Therefore, our findings suggest a role for these biomarkers as supportive diagnostic tools during the emergence of ARDS.

Development of ARDS in a population at risk depends on both genetic predisposition and interaction between several biological pathways, including inflammation, coagulation, and fibrinolysis (19). We intentionally observed patients in the ICU for 1 week after inclusion for biomarker detection. This was inspired by observational studies suggesting that the majority of patients are identified with ARDS within 72 h of the recognition of risk factors, and nearly all within 7 days of hospital stay (22, 25, 26). However, in spite of our efforts to keep low tidal volumes and to titrate PEEP to a point above the lower inflection of the transpulmonary pressure–volume curve, yielding optimal oxygenation, both tidal volume and plateau pressure increased significantly at *T*_7_, and we were not able to prevent PaO_2_/FiO_2_ from falling in the ARDS group. This is consistent with the derangements in oxygenation and lung mechanics in developing ARDS, as reported by other researchers (2, 14).

### ARDS and Biomarkers of Coagulation and Fibrinolysis

Investigators have focused on different biomarkers of ARDS (3, 5, 19, 24), but so far, without significant preferences. Protein C plasma levels are reduced in patients with severe sepsis (26) and in victims of ARDS, due to a greater consumption of this anticoagulant and anti-inflammatory protein (19, 20). However, when it was discovered that replacement therapy with recombinant human activated protein C had no significant effect on survival, neither from severe sepsis nor from ARDS, the interest in protein C vanished (27, 28). Alterations in plasma coagulation and fibrinolysis have not yet been evaluated in view of the latest diagnostic criteria of ARDS that define “acute onset” as a condition developing within 1 week of a known insult, or a new or worsening respiratory failure (29).

We included patients during a 1-year period since some of the conditions with increased risk of ARDS, like influenza and pneumonia have their seasonal peaks. To detect a possible significance of plasma concentrations of TF, PAI-1, and t-PA, as diagnostic markers, we studied an ICU population, which had increased risk of ARDS and required mechanical ventilation for at least 24 h. Several researchers have suggested a potential association between TF and the occurrence of ARDS. TF appears to play a pivotal role both in regulating endothelial permeability and in activating the external coagulation pathway in severe infections (5, 30, 31). Moreover, investigators demonstrated higher plasma TF levels in parallel with lower PLTs and higher incidence of DIC in patients with ARDS, in whom the primary injury was trauma or sepsis (30). Recently, Xue and co-workers found that, patients with sepsis-induced ARDS had significantly higher levels of TF on the day of admission as compared with non-ARDS patients. The AUC for the diagnosis sepsis-induced ARDS was 0.749. These investigators concluded that TF is an independent predictor of 30-day mortality in patients with severe sepsis (OR 1.41; 95% CI 1.24–1.69) with AUC 0.718 (5). Consistent with the latter workers, we showed that TF increased significantly the seventh day after inclusion in the ARDS group as compared with non-ARDS patients (Figure 2A). A ROC curve analysis displayed a sensitivity of 71% with AUC of 0.78, *p* = 0.02, which indicates a fair test value in favor of diagnosing ARDS, according to the published criteria (2, 5). However, we did not observe any correlation between TF and DIC, since none of our patients was diagnosed with that condition. In contrast, TF plasma levels correlated moderately with ventilation parameters (Figures 3B,C), most likely, because of a high proportion of patients with pneumonia (42%) in the ARDS group. Therefore, we consider TF to be a tool for supporting ARDS diagnosis, but without any predictive value since the increase in plasma concentration occurred after 1 week of ICU stay.

In the present study, PAI-1 plasma concentration also increased significantly in ARDS patients on the seventh day of stay in ICU (Figure 2B). The rise in PAI-1 correlated slightly with a parallel increase in TF (Figure 3A) and ROC curve analysis (Figure 4) revealed a sensitivity of 64% with AUC of 0.775; *p* = 0.024, which indicates a somewhat poorer test in favor of diagnosing ARDS, as compared with the corresponding test of TF. Previous investigators have noticed that ARDS patients exhibit early elevation of PAI-1 activity in both plasma and air spaces in concert with a decrease in alveolar fibrinolysis (32, 33). Thus, El Solh and co-workers showed that tracheal aspirate PAI-1 antigen levels, sampled 8 h after witnessed aspiration, increased by a fivefold in those who progressed to ARDS, as compared with those with uncomplicated aspiration pneumonitis (34). The reason for the discrepancy between our results and those of the latter researchers, aside from different analytic techniques, could be that changes in tracheal aspirate PAI-1 antigen concentration occurs earlier as compared to the changes in plasma concentration (32).

Lately, Sapru and co-workers demonstrated in pediatric patients, that increased plasma concentrations of PAI-1, as determined the day after diagnosing ARDS, were associated with increased mortality and fewer ventilator-free days until day 28 (24). Furthermore, investigators who showed that plasma concentrations of PAI-1 increased from days 0 to 3 after diagnosing ARDS, predicted death with an odds ratio of 1.66 (*p* = 0.006) (20). Moreover, a prospective study of 50 patients with ARDS at an early stage showed significantly higher PAI-1 levels in those who died as compared with those who survived (19). However, we confirmed none of the latter findings in this pilot study, which was underpowered for such investigations.

As an acute-phase protein, the serine protease inhibitor PAI-1 binds covalently to t-PA, reduces formation of plasmin, and impedes fibrinolytic bleeding. Moreover, recent experimental studies indicate that PAI-1 also plays a role in upregulating inflammation after exposure to lipopolysaccharides or avian flu virus in conditions like ARDS (35, 36). However, in the present study, when we compared the ARDS group with non-ARDS patients, we found no significant differences in t-PA levels during the first week after inclusion. Thus, whether t-PA is a predictive marker of ARDS is still not concluded, but this hypothesis was not supported in the present prospective pilot study.

We admit that our study has several limitations because of the small number of patients included. For this reason, we could not expect to find significant differences between groups neither in ICU stay and mechanical ventilation, nor in mortality rates. Despite the fact that we found a lower plasma concentration of t-PA at *T*_3_ in ARDS survivors in comparison with non-survivors, the test had a power of only 60% due to low sample size. However, by keeping the ratio between the groups unchanged, an increase in sample size to 14 survivors and 6 non-survivors, would give a significant difference with a power of 80%. Correspondingly, to reach a significant intergroup difference in t-PAI with the same power at *T*_7_, would require a total sample size of 317 patients. Therefore, due to low sample sizes, we were not able to strengthen our findings by means of multivariate analysis. Since mean time to diagnosing ARDS was on the average 3 days, we also admit as a limitation of any predictive role, the fact that most of the patients already had ARDS at the time of plasma sampling at *T*_3_, whereas significant changes in TF and PAI-1 occurred first at *T*_7_. Nevertheless, our observations indicate a role for TF and PAI-1 as tools for supporting ARDS diagnosis, although significant changes occurred relatively late in the evolution of disease.

We also denote as a weakness of this study that the ARDS patients incorporated many serious risk factors and comorbidities when admitted to the ICU. Therefore, those who were included had different lengths of hospital stays before admission to the ICU. However, the prior history was not an issue for evaluation in the present study.

According to ARDS definition (2), we should be able to identify patients with mild ARDS that potentially correlates with a better prognosis. However, besides a low number of patients included, we did not follow oxygenation variables often enough to be able to evaluate them properly against changes in ventilator settings, or other variables on an hourly basis. For the same reason, changes in concentrations of biomarkers that might have taken place, between the days of plasma sampling remain undiscovered. We also realize that sampling of bronchoalveolar lavage fluid, in parallel with the plasma samples, could have strengthened the findings, and maybe enabled the discovery of changes in biomarkers at an earlier time point.

A limitation is also the course of ARDS development that may influence the changes in biomarkers and outcome significantly. We included only patients with high risk of developing ARDS, but even among them, particularly patients with severe pneumonia have a poor prognosis in comparison with victims of multi-trauma and/or those undergoing massive blood transfusions. Finally, due to limited sample sizes, the findings of this pilot study need confirmation in a larger randomized controlled multicenter trial.

## Conclusion

Fourteen of the 24 patients on mechanical ventilation whom we suspected to be at increased risk, developed ARDS on the average 3 days after admission to the ICU. Activation of the coagulation and the fibrinolytic systems in terms of increased plasma levels of TF and PAI-1 occurred on the seventh day of ICU stay in patients diagnosed with ARDS, thus indicating a supportive diagnostic role for these biomarkers. Our findings need confirmation in a larger randomized controlled multicenter trial.

## Author Contributions

All authors have contributed substantially to the study design, analysis and interpretation of data, and drafting or revising the article. AO, MS, and OS conceived the study. MS was responsible for data collection and informed the patients. IV, AS, IJ, and JS performed data analysis, AO and MS made interpretation of data. AO and LB drafted and revised the manuscript. All authors read and approved the final manuscript.

## Conflict of Interest Statement

The authors declare that the research was conducted in the absence of any commercial or financial relationships that could be construed as a potential conflict of interest.
